# Acute biliary pancreatitis caught in the act

**DOI:** 10.1002/ccr3.3343

**Published:** 2020-09-12

**Authors:** Vincent Zimmer

**Affiliations:** ^1^ Department of Medicine Marienhausklinik St. Josef Kohlhof Neunkirchen Germany; ^2^ Department of Medicine II, Saarland University Medical Center Saarland University Homburg Germany

**Keywords:** bile duct stone disease, biliary pancreatitis, endoscopic retrograde cholangiopancreatography, endoscopic ultrasound, stone passage

## Abstract

Endoscopic ultrasound is instrumental in biliary pancreatitis for stratifying patients for invasive ERCP, given high rates of spontaneous stone passage. This is reflected in this report illustrating “triple detection” of stones above, at, and below the papilla.

## Graphical abstract

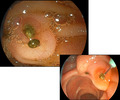

A 90‐year‐old female patient presented with right upper quadrant (RUQ) pain later radiating to the back. Laboratory data confirmed cholestasis (γ‐glutamyl transferase 903 U/L, alkaline phosphatase 438 U/L) complicated by acute biliary pancreatitis (serum lipase 6901 U/L, C‐reactive protein 6.1 mg/dL). Transcutaneous abdominal ultrasound indicated exuberant cholecystolithiasis with an equivocal bile duct diameter of 8 mm. The patient proceeded to endoscopic ultrasound (EUS) indicating distal bile duct stone disease with significant edema in the papillary region.^1^ Duodenoscopy during same‐session endoscopic retrograde cholangiography (ERC) confirmed papillary stone impaction as well as passed stone material. (Figure 1A) We performed primary needle‐knife precut papillotomy (NKPP—compare *inset* in Figure 1A) with a view of the impacted stone showing immediate disimpaction. Likewise, another larger stone of 6 mm could be extracted from the distal bile duct after conventional guidewire‐guided extensional papillotomy. (Figure 1B) The patient took an uncomplicated further clinical course on the normal ward. Same‐admission cholecystectomy was recommended, however, declined by the patient.^2^ This case is uncommon in illustrating stone material above, at, and below the level of the papilla reflecting the dynamics in stone migration in acute biliary pancreatitis.

**FIGURE 1:**
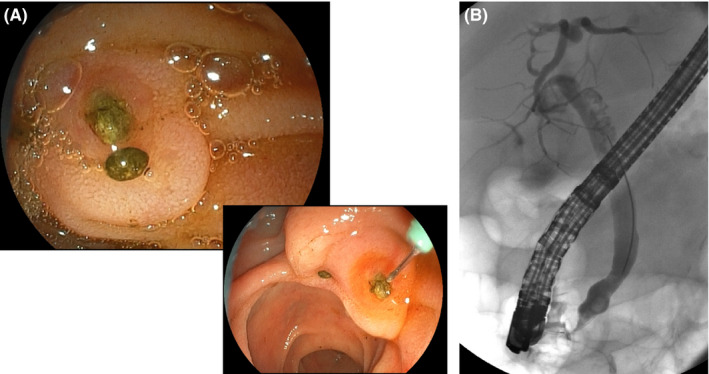
A, Duodenoscopic view of the papilla during endoscopic retrograde cholangiography (ERC) indicating a small, spontaneously passed stone as well as papillary stone impaction treated by primary needle‐knife precut papillotomy (NKPP—compare *inset*) without prior biliary cannulation. B, Cholangiography indicating persistent distal bile duct stone disease being cleared in a conventional fashion after guidewire‐assisted extension papillotomy

## CONFLICT OF INTEREST

None declared.

## AUTHOR CONTRIBUTIONS

VZ: provided clinical care, drafted the manuscript, and finalized and approved the manuscript.

## ETHICAL APPROVAL

Not warranted.
